# Application of Single-Port Laparoscopic Surgery in Myomectomy

**DOI:** 10.3389/fonc.2021.722084

**Published:** 2021-09-24

**Authors:** Lili Jiang, Deming Tong, Yan Li, Qifang Liu, Kuiran Liu

**Affiliations:** ^1^ Department of Obstetrics and Gynecology, Shengjing Hospital of China Medical University, Shenyang, China; ^2^ Department of General Surgery, General Hospital of Northern Theater Command, Shenyang, China

**Keywords:** myomectomy, single-port access (SPA) laparoscopic surgery, three-port laparoscopic surgery, uterine fibroids, gynecological surgery

## Abstract

**Research Question:**

The use of a power morcellator in laparoscopic myomectomy is a controversial topic. The application of single-port laparoscopy solves this problem, but its safety, efficacy and prognosis are also challenges. The purpose of this study was to compare the clinical application of single-port laparoscopy and traditional three-port laparoscopy in myomectomy.

**Design:**

This is a retrospective review of a total of 120 patients who underwent single-port laparoscopic myomectomy (n=60) or traditional three-port laparoscopic myomectomy (n=60), performed between January 2019 to December 2020. The operation time, intraoperative blood loss, specimen removal time, hemoglobin change after operation, postoperative ambulation time, first exhaust time after surgery, the length of hospital stay, pain score on the day, the first day after operation and the satisfaction of abdominal wall scar were evaluated for the surgical outcomes.

**Results:**

Compared with the traditional three-port laparoscopic group, the specimen removal time, postoperative ambulation time, first exhaust time after surgery, the length of hospital stay were all shorter, the satisfaction of abdominal wall scar were higher in single-port laparoscopic group. The duration of surgery was longer in single-port laparoscopic group significantly. The differences were statistically significant (P<0.05). The intraoperative blood loss, hemoglobin change after operation, pain score on the day of operation and the first day after operation of the two groups had no differences (P>0.05).

**Conclusions:**

The clinical effect of single-port laparoscopic myomectomy is satisfactory and can be popularized in clinic.

## Introduction

Uterine myoma is a common benign tumor of female reproductive organs, the overall incidence rate was 40% to 60% at the age of 35 years, 70% to 80% at 50 years of age (1). About 30% of the patients had abnormal uterine bleeding (increased menstruation, secondary anemia) and pelvic compression symptoms (abnormal urination, constipation and diarrhea) (2). A large number of clinical practice shows that the main treatment is still surgery. In recent years, laparoscopic myomectomy has become the first choice because of its less invasive and better surgical outcome (3). Single-port laparoscopic technology has become a hot spot recently, the advantages of it can be maximized by reducing postoperative pain and improving aesthetics. However, the feasibility and safety of it has not been determined. This study aims to explore the feasibility of single-port laparoscopic myomectomy in the field of gynecology by studying the efficacy and recovery of patients with uterine fibroids undergoing single-port laparoscopic myomectomy and traditional three-port laparoscopic myomectomy.

## Methods

### General Information

This study selected 120 patients with uterine fibroids who underwent laparoscopic myomectomy in the gynecologic department of Shengjing Hospital of China Medical University from January 2019 to December 2020. The medical information of each patient was reviewed retrospectively. The inclusion criteria: ①All patients were diagnosed as hysteromyoma by pelvic ultrasound and pelvic MR before operation. ②The patients had signed the informed consent. ③The umbilicus is normal. Exclusion criteria: ①Conversion to open surgery or other surgical methods (Massive bleeding occurred during laparoscopic surgery, and it was impossible to stop bleeding under the laparoscopy; It was found that the anatomical structure was unclear and the adhesion was serious during the operation, which made the laparoscopic surgery could not be carried out; Intraoperative frozen pathology showed malignant transformation of hysteromyoma,etc). ②Malignant transformation of uterine fibroids. ③Submucosal fibroids. ④Severe medical system diseases (heart, lung, brain, liver, kidney and other organ abnormalities). The patients were randomly divided into the observation group and the control group (60 cases in each group) according to the different operation methods. The patients in the observation group were treated with single-port laparoscopic surgery, and the patients in the control group were treated with traditional three-port laparoscopic surgery. Postoperative pathology was uterine leiomyoma. There were no significant differences in age, pregnancy, birth and other general conditions between the two groups. The study was approved by the China Medical University Research Ethics Committee. All of the operations were performed by surgeons experienced in laparoscopic surgery.

### Surgical Procedure

**Figure 1:**
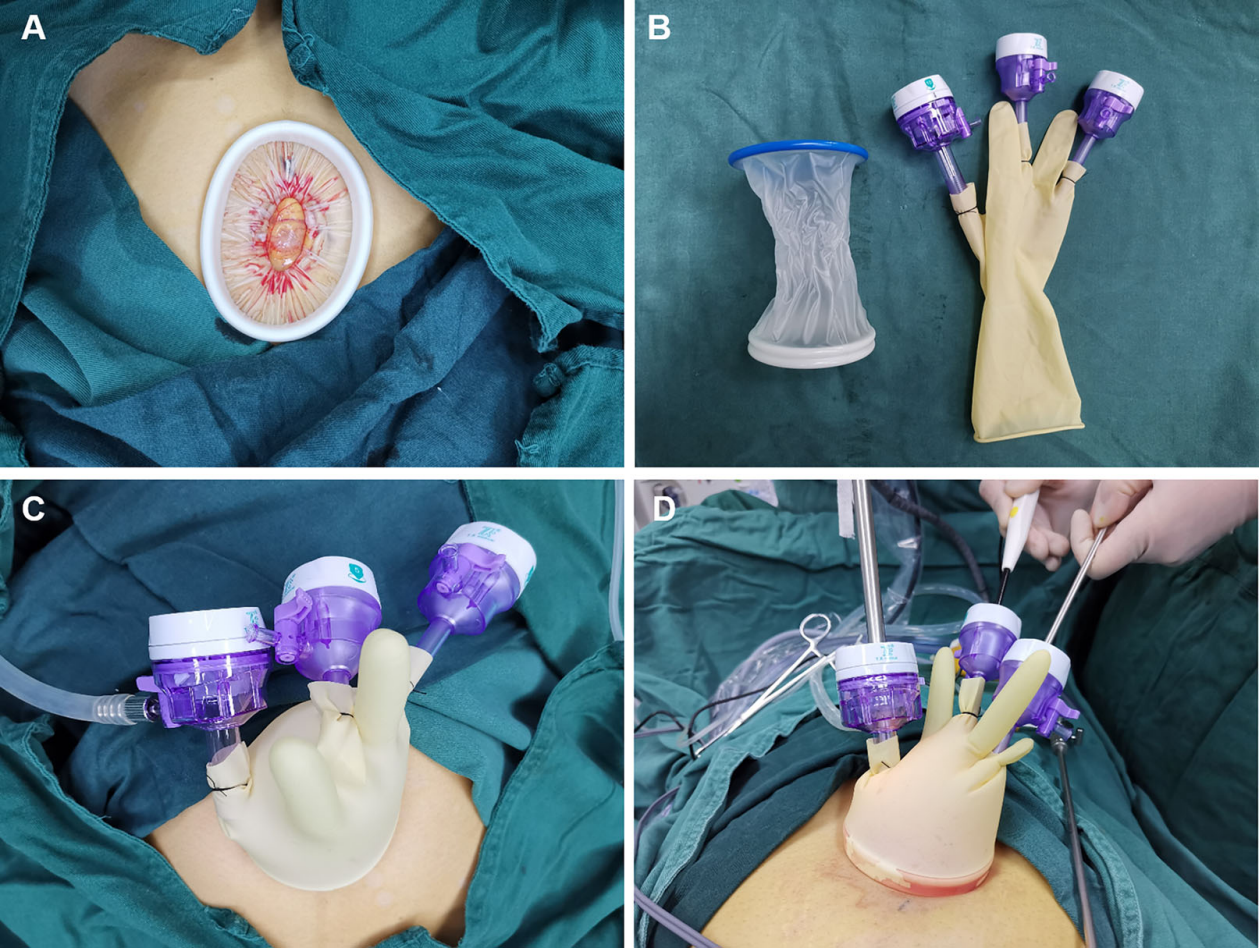
**(A)** The disposable incision protection sleeve (Lookmed, Jiangsu, China) was placed in the incision. **(B)** Single-port laparoscopic approach connection instrument. **(C)** Self-made simple single-port laparoscopic access. **(D)** The instruments enter the abdominal cavity through single-port laparoscopic access.

All patients in the two groups received standardized preoperative nursing preparation and general anesthesia. Single-port laparoscopic surgery was performed using the following techniques. After partial eversion of the umbilicus, a 2-3cm longitudinal incision was made at the umbilicus. The umbilical incision was lifted, the skin and subcutaneous tissue were incised layer by layer, and the peritoneum was incised after confirming that there was no intestinal adhesion below the incision. The disposable incision protection sleeve (Lookmed, Jiangsu, China) was placed in the incision, the inner ring was placed in the abdominal cavity, and the outer ring was left to the abdominal wall to form a single-port laparoscopic approach platform (**Figure 1A**). A sterile glove was connected with the outer ring. The thumb of the glove was cut, and 10mm trocar (Dike, Guangzhou, China) was placed as the access of a scope and laparoscopic instruments. In order to prevent air leakage and loosening at the joint, No. 7 silk thread was used to fix and tie tightly, and the 5mm (Dike, Guangzhou, China) trocars were inserted into the other two fingers as the instrument port (**Figures 1B, C**). This is a self-made simple laparoscopic single-port (**Figures 1C, D**). The advantage is that it can save the cost for the patients without affecting the operation.

**Figure 2:**
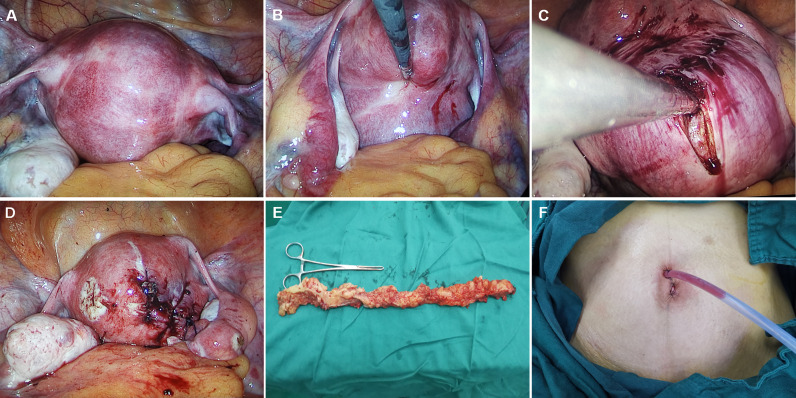
**(A–F)** The operation pictures.

The remaining procedures were the same as those of multi-port laparoscopic surgery. Carbon dioxide was injected at a pressure of 13 mm Hg and a rigid 0° or 30° 5-mm laparoscope was inserted (Karl Storz, tutlingen, Germany). 30° laparoscope is a better choice because it provides a wide field of vision. The presence, location and size of leiomyomas were examined. In order to reduce the bleeding of leiomyoma, the uterus was injected with Pituitrin after dilution, and the blood pressure and heart rate were observed. The myomas were completely removed by longitudinal incision along the capsule of uterine myoma and hemostasis was performed by bipolar electrocoagulation. Uterine fibroids were removed from the umbilicus using endopouch specimen retrieval bag (Wellead, Guangzhou, China). 1-0 absorbable sutures were used for the uterine wound closure. Through the umbilicus, a drainage tube was left in place after surgery. The umbilicus was sutured in three layers. The operation pictures are shown as follows (**Figures 2A–F**).

### Observation Index

①The duration of surgery, specimen removal time, intraoperative blood loss during surgery, Changes of hemoglobin after operation, first exhaust time after surgery, postoperative ambulation time and the duration of the postoperative hospital stay were observed and recorded. ②Visual analogue scale (VAS) was observed and recorded. Mild pain (1-3, slight pain can be tolerated), moderate pain (4-6, pain and affect sleep, can be tolerated), severe pain (7-10, gradually strong pain, unbearable, affect appetite and sleep). ③30 days after operation, the satisfaction of the two groups on abdominal scar was compared. The score was 1-5 according to the wound recovery basing the subjective evaluation of patients after operation. The higher the score, the higher the satisfaction.

### Statistical Analysis

SPSS ver. 26.0 (SPSS Inc., Chicago, IL, USA) was used to perform the Statistical analysis. The p-values less than 0.05, as calculated using the chi-square test or Fisher’s exact test, were considered to indicate statistical significance.

## Results

The mean age of the women who accepted single-port laparoscopic myomectomy was 38.1 years (range, 25–47 years). Their average number of gravidity and parity were 1.57 ± 1.33 and 0.83 ± 0.65. The number of previous abdominal operations was 0.70 ± 0.75. There was no significant difference between single-port laparoscopic group and traditional three-port laparoscopic group in age, pregnancy, labor and other general conditions (**Table 1**).

**Table 1:** Clinical characteristics of the patients in single-port laparoscopic group and traditional three-port laparoscopic group.

Characteristics	Single-port laparoscopic group (n = 60)	Traditional three-port laparoscopic group (n = 60)	t/χ^2^	P Value
**Age**	38.10 ± 6.47	37.17 ± 6.54	0.556	.580
**Gravidity**	1.57 ± 1.33	1.53 ± 1.48	0.092	.927
**Parity**	0.83 ± 0.65	0.63 ± 0.56	1.283	.205
**No. of previous abdominal operations**	0.70 ± 0.75	0.53 ± 0.57	0.968	.337

Surgical outcomes are shown in **Table 2**. During the surgery, the mean number of myomas resected by myomectomy and size of myomas in single-port laparoscopic group were 1.70 ± 1.02 and 7.85 ± 3.10cm which had no differences with the traditional three-port laparoscopic group. The surgical outcomes were not affected by the size and number of myomas. The operation time in single-port laparoscopic group was 80.67 ± 18.37 minutes, which was longer than the traditional laparoscopic group(P<0.05). The specimen removal time was 1.70 ± 0.65 minutes, which was shorter than the traditional laparoscopic group(P<0.05). After operation, the ambulation time and first exhaust time were 12.65 ± 4.32 hours and 27.70 ± 9.14 hours in single-port laparoscopic group. Calculated (P<0.05), indicating a statistically significant difference with the traditional laparoscopic group. There was no extension of the postoperative hospital stay in single-port laparoscopic group. On the contrary, the postoperative hospital stay was shortened(P<0.05). The intraoperative blood loss of single-port laparoscopic group was 57.00 ± 35.44 ml, which was not significantly different from that of the 54.67 ± 34.01 ml in traditional laparoscopic group (P>0.05). The hemoglobin change after operation was 19.00 ± 10.44g/L in the single-port laparoscopic group which was not significantly different between the two groups.

**Table 2:** Surgical outcomes of the patients in single-port laparoscopic group and traditional three-port laparoscopic group.

	Single-port laparoscopic group (n = 60)	Traditional three-port laparoscopic group (n = 60)	t/χ^2^	P Value
**No. of myomas resected by myomectomy**	1.70 ± 1.02	1.86 ± 1.55	-0.492	.624
**Size of myomas (cm)**	7.85 ± 3.10	7.40 ± 2.03	0.665	.509
**Operation time (min)**	80.67 ± 18.37	70.47 ± 17.11	2.225	.030
**Blood loss (ml)**	57.00 ± 35.44	54.67 ± 34.01	0.260	.796
**Specimen removal time(min)**	1.70 ± 0.65	3.27 ± 0.58	-9.815	.000
**Hemoglobin change after operation(g/L)**	19.00 ± 10.44	19.87 ± 10.18	-0.326	.746
**Postoperative ambulation time(h)**	12.65 ± 4.32	15.12 ± 4.83	-2.089	.041
**First exhaust time after surgery(h)**	27.70 ± 9.14	33.16 ± 5.31	-2.833	.006
**Postop. hospital stay (days)**	4.03 ± 0.99	4.67 ± 0.88	-2.600	.012

After operation, we evaluated the pain score of the two groups and the follow-up of scar satisfaction 30 days after operation to evaluate the operation effect. The data were shown in **Table 3**. Visual analogue scale (VAS) was observed and recorded to evaluate the pain score. Mild pain (1-3, slight pain can be tolerated), moderate pain (4-6, pain and affect sleep, can be tolerated), severe pain (7-10, gradually strong pain, unbearable, affect appetite and sleep). On the day of the operation, the VAS in the single-port laparoscopic group 4.13 ± 0.63. On the first day after operation, the VAS was 3.07 ± 0.89 in the single-port laparoscopic group. There were no statistical differences between the single-port laparoscopic group and traditional laparoscopic group(P>0.05). 30 days after operation, the satisfaction of the two groups on abdominal scar was compared. The score was 1-5 according to the wound recovery basing the subjective evaluation of patients after operation. The higher the score, the higher the satisfaction. The score of the single-port laparoscopic group was 4.17 ± 0.46 which was higher than the traditional laparoscopic group(P<0.05).

**Table 3:** Comparison of VAS score and abdominal scar satisfaction score of each group.

	Single-port laparoscopic group (n = 60)	Traditional laparoscopic group (n = 60)	t/χ^2^	P Value
**VAS score on operation day**	4.13 ± 0.63	4.03 ± 0.85	0.518	.606
**VAS score on the first day after operation**	3.07 ± 0.89	2.93 ± 0.58	0.698	.488
**Abdominal scar satisfaction score**	4.17 ± 0.46	3.47 ± 0.63	4.917	.000

## Discussion

Uterine myoma is a very common disease of female reproductive system. The pathogenesis is related to many factors, such as race, age, menarche age, genetic factors and so on (4). Many myomas are asymptomatic, about 30-40% of cases can show different symptoms, depending on the location and size of myoma, which can lead to abnormal menstruation, dysuria, abortion, infertility and so on (5). In previous epidemiological studies, the prevalence of uterine myoma was underestimated as the majority of studies focused on symptomatic women (6) Menorrhagia can be secondary to anemia, and even life-threatening (7). In previous epidemiological studies, the prevalence of leiomyoma was underestimated because the majority of studies focused on symptomatic women (8). Myomectomy is a common surgical method for the treatment of uterine fibroids. The choice of treatment depends on the age of the patient and the desire to preserve fertility or avoid radical surgery (9). With the increase of marriage age and the increase of infertility associated with myomas, this kind of operation method has increased (10). In recent years, minimally invasive surgery has been widely used in the field of gynecology. A meta-analysis of 576 cases compared transabdominal myomectomy with laparoscopic myomectomy showed that laparoscopic myomectomy had faster recovery, less intraoperative blood loss, less postoperative pain, and lower overall complications, which was a better choice than open surgery (11). Laparoscopic myomectomy is considered more difficult by many gynecologists, but its advantages are real, and there is no difference in reproductive outcomes compared with traditional open surgery (2, 12). With the continuous development of minimally invasive concept, it is the pursuit of surgeons to minimize surgical trauma and achieve the best cosmetic effect. Single-port laparoscopic surgery has become a hot spot because it uses the natural pores of the navel to hide the surgical incision and has the characteristics of beautiful incision and fast postoperative recovery.

In our study, we compared the effect of single-port laparoscopy and traditional three-port laparoscopy in myomectomy. Compared with the control group, the specimen removal time, the first exhaust time after surgery, the postoperative ambulation time and the Postoperative hospital stay in the observation group were shorter, and the differences were statistically significant (P<0.05). It showed that single-port laparoscopy had the characteristics of less trauma and faster recovery. This was consistent with the research results of You et al (13) that compared with traditional laparoscopy, single-port laparoscopic surgery didn’t lead to prolong suture time and hospital stay. As the incision at the belly button can reach 3cm after being opened, the specimen is easier to take out which makes the single-port laparoscope having more advantageous. Postoperative pain is an important indicator that affects the patient’s recovery, not only affecting the patient’s time to get out of bed after surgery, but also causing a serious psychological burden on the patient. In our study, the VAS score on operation day was 4.13 ± 0.63 and the VAS score on the first day after operation was 3.07 ± 0.89. The VAS scores of the single-port laparoscopic group had no difference with traditional laparoscopic group. It indicated that although the umbilical incision of single-port laparoscopic surgery was larger relatively, it did not increase the postoperative pain of the patients. In a follow-up study of patients’ satisfaction with abdominal scars at 30 days after surgery, the score of the single-port laparoscopic group was higher than those of the traditional three-port laparoscopic group significantly. It showed that single-port laparoscopic surgery uses the skin folds naturally formed in the belly button as a channel, truly achieving “scar-free”, the incision was more beautiful and patients’ satisfaction improved.

In the study, there was no significant difference in intraoperative blood loss and postoperative hemoglobin changes between the observation group and the control group, suggesting that single-port laparoscopic myomectomy didn’t increase the risk of bleeding, and there was no difference in surgical safety between the two groups. The results of this study also showed that the operation time of the observation group was longer than that of the control group (P<0.05). The possible reason was that the operation space of single port laparoscopic surgery was limited, the operation time was short, the operation technology was difficult and more clinical experience was needed. Through skilled operation, the operation time could be shortened (14).

In traditional three-port laparoscopic myomectomy, we used the laparoscopic uterine power morcellation to remove the fibroids. One of the main problems with power morcellation was that the removed tissue spreads to the surrounding area (15). The spread of benign lesions such as leiomyomas can lead to recurrence (16). Paul (17) et al. reported a case of recurrence in a short period of time after laparoscopic myomectomy in a young woman, which was considered to be caused by the residual tumor body during the first operation or the spread of tumor fragments caused by the laparoscopic uterine power morcellation. More worrying is the risk of the spread of malignant tumors, such as endometrial cancer, which may lead to a decline in overall survival rate (18). In 2014, the U.S. FDA issued a statement warning that in order to prevent the damage caused by the spread of unknown uterine fibroids to patients, it opposes the use of uterine power morcellation in laparoscopic hysterectomy or myomectomy (19). It is worthwhile to integrate that the single-port laparoscopic myomectomy procedure avoids the risk of tumor dissemination caused by the traditional three-port laparoscopic uterine power morcellation. During single-port laparoscopic myomectomy, the removed fibroids were placed in the specimen retrieval bag and lifted to the umbilical incision and removed by cold knife, which reduced the risk of fibroids dissemination. The endopouch specimen retrieval bag is an innovative single-use disposable device designed to be used as a receptacle for benign tissue mass during gynecological procedures such as laparoscopic myomectomy or laparoscopic hysterectomy. There is another new type of tissue morcellation bag which can be used in conjunction with the tissue morcellation system to safely contain and remove the shredded benign tissue mass. The device has unique features to allow for quick deployment, insufflation, morcellation and spill-proof withdrawal of the bag. Thereby reducing the patients’ pain and surgical trauma caused by recurring diseases and even life threatening caused by medical dissemination.

Single-port laparoscopic surgery also has limitations in the treatment of uterine fibroids. Because the relevant instruments can only enter the abdominal cavity through the umbilical incision, the instruments are relatively concentrated and interfere with each other. It is difficult to form an operation triangle. The operation lacks a sense of space and three-dimensionality. The difficulty increases in operation and an experienced surgeon is required to perform the operation. The operation time of the single-port laparoscopic group in our study is higher than that of the traditional three-port laparoscopic group, which reminds that the surgeon needs to master skilled techniques and continuously accumulate experience in order to reduce the operation time. Moreover, the conditions of the operation should be comprehensively evaluated, the indications should be grasped and choose a more appropriate surgical method for patients with contraindications before the operation.

## Conclusion

In summary, the use of single-port laparoscopic surgery to treat uterine fibroids can effectively reduce postoperative pain, accelerate postoperative recovery of patients, improve incision beauty and patients’ satisfaction which can be applied to the treatment of uterine myoma and is worthy of promotion.

## Data Availability Statement

The original contributions presented in the study are included in the article/supplementary material. Further inquiries can be directed to the corresponding author.

## Ethics Statement

The studies involving human participants were reviewed and approved by Medical Ethics Committee of Shengjing Hospital of China Medical University. The patients/participants provided their written informed consent to participate in this study. Written informed consent was obtained from the individual(s) for the publication of any potentially identifiable images or data included in this article.

## Author Contributions

LJ conducted main part of the study design and was the major contributor in writing the manuscript. DT was responsible for part of the literature search. YL collected specimens and took the images. QL was responsible for drawing and reviewing pictures. KL was responsible for checking the data, reviewing, and revising the article. All authors contributed to the article and approved the submitted version.

## Funding

This research received the support from “Scientific research funding project of Liaoning Provincial Department of Science and Technology (No.2020JH2/10300050)”.

## Conflict of Interest

The authors declare that the research was conducted in the absence of any commercial or financial relationships that could be construed as a potential conflict of interest.

## Publisher’s Note

All claims expressed in this article are solely those of the authors and do not necessarily represent those of their affiliated organizations, or those of the publisher, the editors and the reviewers. Any product that may be evaluated in this article, or claim that may be made by its manufacturer, is not guaranteed or endorsed by the publisher.
